# The Highways

**Published:** 1893-06

**Authors:** 


### Editorial.

### The Highways.

Not long since there came to hand a petition, the object of which was to secure names to be appended to a memorial which should be presented to Congress, looking to the permanent improvement and betterment of the highways of our country.

It is a noteworthy fact that this subject has in this age of general improvement and new devices received relatively far too little attention; when compared with the other elements and factors of progress in our civilization our highways have fallen greatly behind. The time has arrived when facility and rapidity of transportation and travel are greatly in demand. The present requirements are quite intolerant of the old modes of transportation. The wonder is that long ere this more special attention has not been given to this subject. Rapidity of movement, ease and satisfaction in its accomplishment, and an immense saving in expense, and various resources are attained by the more perfect condition of our highways of travel. All the leading countries of Europe are vastly in advance of the United States in the perfection of their highways and this too when the United States is the richest nation on the globe. It, too, possesses a much greater disposition for improvements and inventions, and an enthusiasm in whatever it undertakes that is not anywhere else excelled. Therefore we conclude that the reason for the present condition of things is that this subject has not been agitated and pressed upon the attention of our people and its importance made known. It is to be hoped that the people of this country will proceed to mend their ways.

